# Comparative Genomic Analysis of Multi-Subunit Tethering Complexes Demonstrates an Ancient Pan-Eukaryotic Complement and Sculpting in Apicomplexa

**DOI:** 10.1371/journal.pone.0076278

**Published:** 2013-09-27

**Authors:** Christen M. Klinger, Mary J. Klute, Joel B. Dacks

**Affiliations:** Department of Cell Biology, University of Alberta, Edmonton, Alberta, Canada; University Of Montana - Missoula, United States of America

## Abstract

Apicomplexa are obligate intracellular parasites that cause tremendous disease burden world-wide. They utilize a set of specialized secretory organelles in their invasive process that require delivery of components for their biogenesis and function, yet the precise mechanisms underpinning such processes remain unclear. One set of potentially important components is the multi-subunit tethering complexes (MTCs), factors increasingly implicated in all aspects of vesicle-target interactions. Prompted by the results of previous studies indicating a loss of membrane trafficking factors in Apicomplexa, we undertook a bioinformatic analysis of MTC conservation. Building on knowledge of the ancient presence of most MTC proteins, we demonstrate the near complete retention of MTCs in the newly available genomes for *Guillardia* *theta* and *Bigelowiella* *natans* . The latter is a key taxonomic sampling point as a basal sister taxa to the group including Apicomplexa. We also demonstrate an ancient origin of the CORVET complex subunits Vps8 and Vps3, as well as the TRAPPII subunit Tca17. Having established that the lineage leading to Apicomplexa did at one point possess the complete eukaryotic complement of MTC components, we undertook a deeper taxonomic investigation in twelve apicomplexan genomes. We observed excellent conservation of the VpsC core of the HOPS and CORVET complexes, as well as the core TRAPP subunits, but sparse conservation of TRAPPII, COG, Dsl1, and HOPS/CORVET-specific subunits. However, those subunits that we did identify appear to be expressed with similar patterns to the fully conserved MTC proteins, suggesting that they may function as minimal complexes or with analogous partners. Strikingly, we failed to identify any subunits of the exocyst complex in all twelve apicomplexan genomes, as well as the dinoflagellate *Perkinsus marinus*. Overall, we demonstrate reduction of MTCs in Apicomplexa and their ancestors, consistent with modification during, and possibly pre-dating, the move from free-living marine algae to deadly human parasites.

## Introduction

The Apicomplexa are a phylum of single-celled eukaryotic organisms of tremendous economic and medical importance world-wide [1]. Members of the genus Plasmodium, including *P. falciparum*, *P. vivax*, *P. malariae*, *P. ovale*, and *P* *. knowlesi* , cause malaria in humans [2]. Despite increased preventive and treatment measures, malaria killed an estimated 1.24 million people globally in 2010, and the world-wide disease burden exceeds 200 000 000 cases per year, with noted implications for poverty (WHO World Malaria Report 2012 [3]). *Toxoplasma gondii* is a ubiquitous parasite; approximately one third of the worldʼs population harbor infections, and seroprevalence rates can be as high as 80% in some countries [4,5]. Infections are usually latent, but can cause severe ocular and neurological disorders in cases of congenital toxoplasmosis and in immunocompromised individuals, especially those with HIV/AIDS [6]. *Cryptosporidium parvum* infects enterocytes and induces diarrhea. Much like *T. gondii*, infection in immunocompetent individuals is not usually fatal; the main burden occurs in conjunction with HIV infection [7]. Other prominent members of the Apicomplexa include *Babesia*, *Theileria*, *Neospora*, and *Eimeria*, all of which are associated with disease in humans and/or livestock [8–11].

These parasites possess divergent versions of many canonical eukaryotic organelles. Although they possess a plastid derived organelle (the apicoplast), it is non-photosynthetic [12]. In most apicomplexans, the Golgi body has aberrant, ring-like morphology [13,14]. Apicomplexa are also the only eukaryotic group with clear mitochondria, but that lack obvious peroxisomes [15]. Additionally, Apicomplexa possess the apical complex, a unique association of cytoskeletal components arranged into a conoid and numerous sub-pellicular microtubules [16]. Associated with this structure are the apical organelles – rhoptries, micronemes, and dense granules. These organelles mediate parasite infection and modification of their target host cells and are essential for parasite viability [17].

Protein targeting to apical organelles is incompletely understood, though many studies have identified key factors. In both *Toxoplasma* and Plasmodium, some transmembrane rhoptry proteins contain canonical lysosome targeting signals in their cytoplasmic portions. These consist of the tyrosine-based YXXϕ and the di-leucine based LL motifs [18,19], which normally mediate targeting to lysosomes and related organelles in an adaptin dependent manner [20]. Studies in *Toxoplasma* demonstrated that targeting of transmembrane proteins containing these domains was AP-1 dependent [21], though this result remains contentious. Additional studies have implicated Rab5A and 5C in microneme protein trafficking and rhoptry biogenesis [22], and homologues of dynamin and sortilin have also been identified as critical for the trafficking of some proteins [23,24]. Microneme protein targeting may also occur through the removal of pro-domains via the action of a Cathepsin-L protease in an intermediate endosomal compartment [25]. Additionally, soluble rhoptry and microneme proteins may also possess multiple independent targeting signals [26], and other pathways have been proposed [27]. The biogenesis and function of these organelles depends on the timely and accurate delivery of protein and lipid components, and although the membrane-trafficking system is clearly involved, the mechanisms and critical components underpinning such processes remain largely unknown (reviewed in [28]).

In general, vesicular traffic requires several basic processes, namely cargo recognition, coat formation, budding/scission, uncoating, delivery and fusion. As would be expected, given the vast array of potential membranous destinations, transport specificity is mediated by contributions from, at least, rabs, SNAREs, coats, Sec1/Munc18-related (SM) proteins, and tethering factors [29,30]. The latter category can broadly be divided into coiled-coil tethers and multi-subunit tethering complexes (MTCs).

MTCs are large heteromeric complexes that display varying subunit number and composition, but can generally be divided into three groups. CATCHR (complexes associated with tethering containing helical rods) consists of the complexes Dsl1, COG, GARP, and exocyst, all of which function in the secretory pathway. HOPS and CORVET are required for endolysosomal transport and form the second group. Finally, the various TRAPP complexes form a group separate from either [31]. Though both coiled-coil proteins and MTCs are capable of tethering vesicles, MTCs are capable of more sophisticated action, and form networks of interactions between rabs, SNAREs, coat proteins, and specific lipids to mediate fusion [32].

Despite the need for complex membrane trafficking in the Apicomplexa, several studies to date have catalogued the absence of numerous core trafficking factors. Although evidence suggested the potential involvement of adaptor proteins (AP) in microneme and rhoptry biogenesis and function, a previous comparative genomic assay identified a complete lack of the AP-3 complex in Babesia, Theileria, and *Cryptosporidium* [33]. Furthermore, *P. falciparum*, *T. gondii*, and *C. parvum* lack any subunits of the ESCRT0, -I, and -II complexes, and, together with *T* *. parva* , lack several subunits of the ESCRTIII-associated complexes [34]. The number of rabs encoded in any apicomplexan genome ranges from eight for *C. parvum* to 15 for *T. gondii* and Neospora [35], as compared to the core ancestral eukaryotic complement of between 19-23 rab subfamilies [36].

A previous study indicated that the MTCs are widely conserved in eukaryotes and thus ancient components of the membrane-trafficking system. However, the only apicomplexan genomes analyzed, *T. gondii* and *P. falciparum*, encode very few MTC components outside of TRAPPI and HOPS [37]. Prompted by the increasingly important role MTCs appear to play in trafficking, and by the lack of associated in vitro and in vivo studies in Apicomplexa, we undertook a comparative genomic analysis of all known MTC components to investigate the complements and evolutionary patterns of these components in apicomplexan genomes. Before undertaking this specific analysis, we first incorporated newly available genomes of highly relevant sister taxa in order to establish baseline subunit retention in the hypothetical apicomplexan ancestor. In addition to these organisms, genomes from organisms representing the breadth of eukaryotic diversity were searched for recently described additional MTC subunits in order to deduce their presence or absence in the Last Eukaryotic Common Ancestor (LECA), and hence, whether they are predicted to be present in a given set of extant eukaryotes, including Apicomplexa.

In this study we find that Apicomplexa have reduced their MTC complement in nearly every case, in two instances losing complexes entirely. Intriguingly, the reduced complexes do appear to be expressed, suggesting the presence of either minimal functional complexes, or analogous subunits, which may confer apicomplexan-specific functions. These findings have implications both for apicomplexan biology, as well as the evolution of MTCs across eukaryotes.

## Methods

### Comparative Genomics

Over the course of our analyses, we utilized a total of 60 eukaryotic genomes, available publically via numerous online databases listed in Table S1. Several genomes used in this study are publically available pre-publication. In the case of *Toxoplasma gondii*, *Neospora* *caninum* , and *Eimeria tenella*, these are available via ToxoDB [38]. In the case of *Cryptococcus neoformans*, Batrachochytrium dendrobatidis, *Bigelowiella* *natans* , *Emiliania* *huxleyi* , and *Guillardia* *theta* , these sequence data were produced by the US Department of Energy Joint Genome Institute (http://www.jgi.doe.gov/) in collaboration with the user community. The genomes of Allomyces macrogynus, *Spizellomyces* *punctatus* , Capsaspora owczarzaki, and Thecamonas trahens are available as part of the Broad Institute’s UNICORN initiative [39].

The following taxa were specifically analyzed in the context of the Vps3/39 analysis but were not included in the larger pan-eukaryotic survey as redundant to included genomes: Allomyces macrogynus, *Aspergillus fumigatus*, *Neurospora crassa*, *Phanerochaete chrysosporium*, *Schizophyllum* *commune* , *Spizellomyces* *punctatus* , *Yarrowia lipolytica*, *Candida albicans*, *Candida* *glabrata* , *Ashbya* *gossypii* , *Amphimedon* *queenslandica* , Thecamonas trahens, and *Trichoplax* *adhaerens* .

Preliminary homology searching was carried out using the BLASTp algorithm [40]. Though we retain the use of yeast nomenclature in figures for consistency, starting queries were either those identified in *Saccharomyces cerevisiae* or *Homo sapiens*. This prevented an arbitrary choice of starting query, and used sequences corresponding to proteins studied in vitro/vivo. This latter distinction decreased the reliance of the assay on purely bioinformatic methods by starting with structurally and functionally defined entities. In either case, the starting proteins were used as initial queries to BLAST the genomes of each organism for potential homologues. All BLAST searches were run online using an embedded tool in the respective database website. Hits scoring an E value of 0.05 or lower were subjected to verification by reciprocal BLAST analysis. This involved the use of candidate proteins as queries in BLASTp searches against the original queries’ (*S. cerevisiae* or *H. sapiens*) non-redundant protein database at NCBI. Only proteins that retrieved the original query sequence, or named homologues thereof, first were deemed truly homologous. These proteins were also subjected to manual inspection to deduce the more subjective aspects of homology, such as size and the presence of conserved domains, as deduced by the CDD program at NCBI [41]. In cases where no candidate proteins scored better than the cutoff value for the initial BLASTp search, the two best matches were still subjected to analysis as above in an effort to identify divergent homologues.

To confirm the initial BLASTp results, we constructed Hidden Markov Models (HMMs). Sequences corresponding to the *H. sapiens*, *S.* cerevisiae, and *Arabidopsis thaliana* homologues of each respective protein, together with homologues of each protein in the genomes searched, were aligned using MUSCLE [42]. HMMs were created from the alignments, and then searched using HMMer v3.0 [43] via a local server. In organisms lacking subunits on the basis of BLAST analysis, any hits using HMMer with an E value less than or equal to 0.05 were subjected to reciprocal BLAST analysis as above. Organisms with putative homologues were compared to the HMMer results to determine if the same protein(s) were retrieved.

If putative homologues could still not be identified, tBLASTn searches were carried out for that organism using the closest related homologous sequence identified in the dataset. These searches were carried out against all genomic sequences in order to identify instances where sequences were present, but not identified as putative transcripts/proteins. Identical cutoff and reciprocal best-hit criteria were utilized as per the BLASTp analysis.

The initial analyses of the *Guillardia* *theta* CCMP2712 and *Bigelowiella* *natans* CCMP2755 genomes were performed while these were still proprietary datasets generated by the Joint Genome Institute. Initial BLASTp searches were completed using *Homo sapiens* and *Arabidopsis thaliana* queries and the “v1 filtered proteins” databases. Candidate homologues with E-values less than or equal to 0.05 were subject to reciprocal BLASTp searches, as described above. In cases where no candidate homologues were identified, BLASTp searches were completed using the “v1 all proteins” databases and tBLASTn searches using the “v1 nuclear scaffolds” and RNA-Seq contigs. HMMer v3.0 searches were completed using Hidden Markov models built with at least one query from each eukaryotic supergroup and the “v1 all proteins” datasets, in cases where homologues were not identified using BLAST. Gene models for G. theta and *B* *. natans* homologues were manually inspected for percent length compared to *H. sapiens* and *A. thaliana* queries, and presence of start and stop codons. New gene models were created as needed.

Additionally HMM profile to profile searches were undertaken. Alignments were built for two sets of sequences. The first set included all identified homologues from all organisms save the query genomes *H. sapiens* and *S. cerevisiae*, and the second set included only homologues from apicomplexan genomes. In both instances, where three or more homologues were identified for a single subunit, only the two with the highest forward and reverse BLAST E values were used, to prevent skewing the resulting HMM towards sequences from one organism by sheer volume of included sequences. These alignments were run through the program HHsearch [44] against clustered versions of the non-redundant (NR) database from NCBI, and the full UniProt database, available as of 2012. Output was analyzed to deduce whether the input alignment contained sequences with homology to clusters of known homologues, utilizing a simple top hit criteria with E value of less than or equal to 0.05. Of 42 queries, 26 retrieved the correct cluster in both databases according to the criteria outlined. An additional ten queries were confirmed by searches against one database but not the other. Six queries (Trs85, Trs130, COG7, COG8, Tip20, and Sec39) were not validated by searches against either database. However, as these queries were identified by the analyses described above, we tentatively retained them for further analysis. Repetition of the analysis utilizing only apicomplexan sequences yielded similar results (Table S2), suggesting the homologues identified are genuine. Importantly, the profile to profile HMM analysis was mainly intended to identify candidate sequences that might have been missed by the less sensitive methods of BLAST or HMMer: no additional subunits were identified in the course of either profile to profile HMM analysis performed.

The results of homology searches are shown in Coulson plot format. These figures were created using automated image generation software [45], followed by subsequent manual additions and editing in Adobe Illustrator.

### Phylogenetic analysis

In the case of some subunits, phylogenetic analyses were performed in order to confirm, and extend upon, the homology searching results. Relevant sequences were aligned using MUSCLE, followed by manual inspection and trimming of the alignments to remove regions of ambiguous nature. The final alignments were then analyzed by Prot-test v3.2.1 to select for the optimal model of sequence evolution [46]. All alignments are available upon request. Mr. Bayes v3.2.1 was used to determine the best tree topology and associated posterior probability values [47]. One million generations were used, with the plateau found by graphical representation and the burn-in value calculated to remove all sub-optimal trees. PhyML v3.0 and RAxML v7.2.6 were used for maximum likelihood bootstrapping, each based on 100 pseudo-replicate data sets [48,49]. Trees were viewed using Fig Tree v1.2 and manually edited using Adobe Illustrator. All phylogenies show the best Bayesian topology and give associated support values for clades reconstructed by all three methods in the order MrBayes/PhyML/RAxML. For all minor internal nodes, these values are replaced by symbols denoting ranges of support, as defined in the figure legend.

### Analysis of expression data

Gene expression data was obtained through EuPathDB, based on RNA-seq and microarray data from multiple experimental sources [50–52]. This raw expression data was transformed to log_2_ values and then plotted as a function of time. In all cases, expression data was consistent among subunits of each complex such that averaging across complex subunits did not dramatically change the expression profile. Therefore, we took the average complex expression at each time point to be the arithmetic mean of the expression of that complex’s subunits. These values were plotted against the time course of each individual experiment to produce four independent graphs of MTC expression in *P. falciparum* and *T. gondii* at different life cycle points.

## Results

### The marine algae *G*. *theta* and *B* *. natans* possess a near complete canonical set of MTC components

In order to understand the evolution of the MTC complement in Apicomplexa, it is important to have a solid assessment of the MTC complement in the ancestral lineage, giving rise to these organisms. Previous comparative genomic analyses to determine the diversity and conservation of the MTC components sampled a taxonomically broad range of eukaryotic genomes. For the majority of the tethering factors under study, their distribution across eukaryotes established their presence in the LECA, and hence provides a null hypothesis for the presence in the genomes of all extant eukaryotes as well [37]. However, due to the unavailability of genome sequences, some of the taxa key to deducing the MTC complement in the nodes leading up to the apicomplexan ancestor, were not sampled. Particularly important are the genomes of the cryptophyte alga *G. theta* and the rhizarian alga *B* *. natans* . The latter is crucial, being the first genome available for the Rhizaria, the third member of the SAR supergroup to which the Apicomplexa belong [53]. We identified a near complete set of all seven of the MTCs in both *B* *. natans* and *G. theta* (Figure S1). This canonical complement is consistent with the sophisticated nature of the cell biological machinery reported to be encoded in these two genomes [54].

### Comparative genomics of newly discovered MTC components

**Figure 1:**
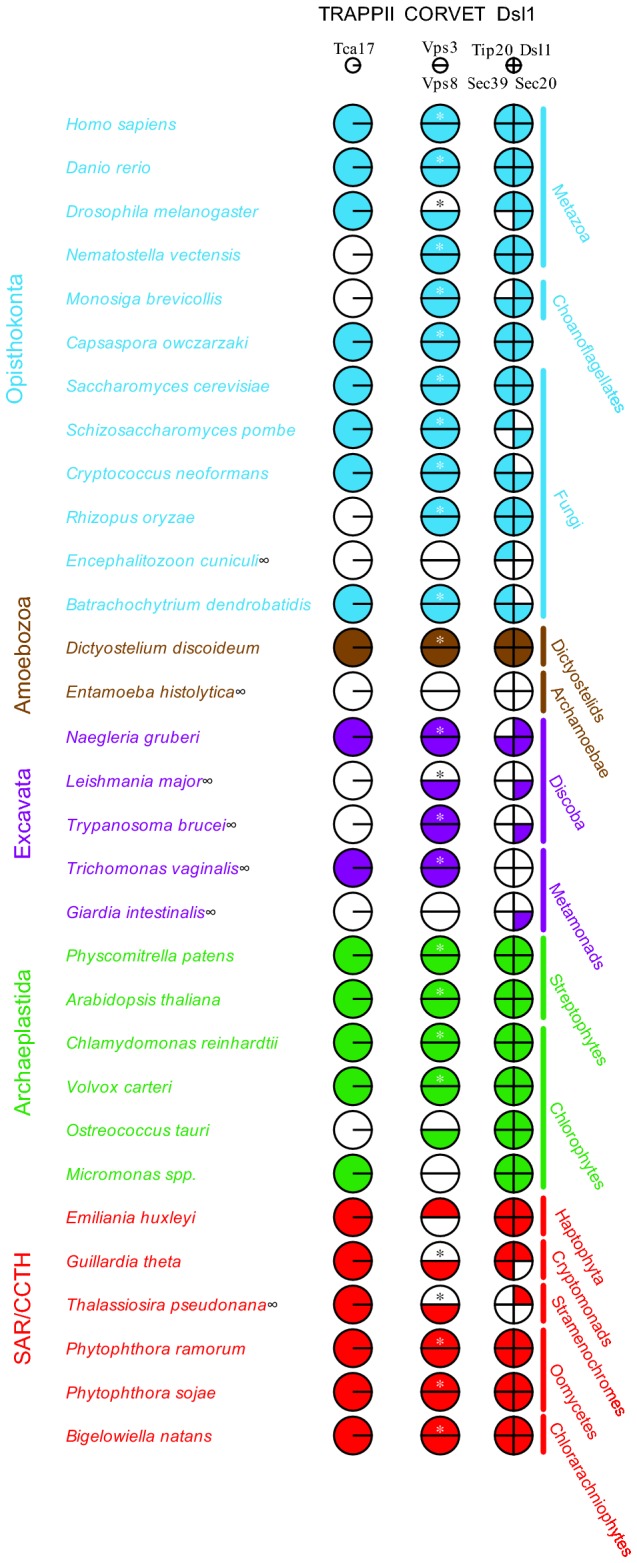
Comparative genomic survey of selected MTC proteins across the diversity of eukaryotes. The analysis demonstrates the conserved nature of the newly described TRAPPII subunit Tca17/TRAPPC2L, the CORVET complex, and the Dsl1 complex. In this and subsequent Coulson plots, filled pie sectors indicate an identified homologue, while unfilled indicate that no homologue was identified. Asterisks indicate that Vps39 is also present in these taxa (See Table S2). Infinity symbols represent organisms with divergent peroxisomes, or in which peroxisomes have not been identified. Colour-coding is arbitrary and for visual purposes only.

Several new subunits of the MTCs have been recently discovered whose evolutionary distribution has not been examined. To provide a solid starting point for investigations into apicomplexan MTC evolution, we undertook a comparative genomic analysis to establish the presence or absence in the LECA of these subunits, by searching 31 genomes covering the breadth of eukaryotic diversity (Figure 1). Specific information regarding the identities and characteristics of all subsequently described proteins are summarized in Table S2.

TRAPPII mediates tethering at the late Golgi, and within the Golgi complex itself [55]. Trs65 and the recently described Tca17/TRAPPC2L proteins are components of the TRAPPII complex in yeast with potentially overlapping functions. Trs65 allows for the oligomerization of TRAPPII complexes, and Tca17, together with Trs33, act to stabilize these oligomers [55]. Given that Trs65 is conserved only among fungi [37], and that mammalian TRAPP complexes are not organized identically to yeast [56,57], we postulated that Tca17 may represent a more general TRAPP subunit. Indeed, we identified genes encoding Tca17 in 22 of the 31 genomes, with at least one homologue present in an organism from each of the six eukaryotic supergroups. Hence, Tca17/TRAPPC2L represents an ancient member of the TRAPP complex and likely has a conserved role in its structure and function.

The Dsl1 complex, in yeast, tethers incoming Golgi vesicles at the ER and also plays a role in peroxisome biogenesis from the ER [58]. In humans, the NRZ/Syntaxin 18 complex subunits are homologous to the yeast Dsl1 complex subunits in the form Zw10/Dsl1, RINT-1/Tip20, and NAG/Sec39 [59–62]. In previous analyses of Dsl1 evolution [37], the complex was deduced as ancient but poorly retained across eukaryotes. This was based on use of the yeast homologues as search queries. However, despite numerous studies demonstrating significant structural conservation [63–65], sequence similarity between Zw10/Dsl1 and NAG/Sec39 is low and confined to specific regions of each protein. Indeed, using a simple BLAST search between human and yeast genomes, neither Zw10 nor NAG retrieved their respective yeast homologues first and vice-versa, though RINT1 and Tip20p retrieve each other first with E values in the range of 10^-3^. Hence, we utilized both yeast and human queries in BLAST and HMMer searches across eukaryotes to identify as many putative homologues of the Dsl1/Syntaxin 18 complex as possible. Though the Dsl1 complex interacts with at least three, and as many as five, different SNAREs [66], we included just one – Sec20/BNIP1 – in our study, in order to remain consistent with the previous analysis to which we endeavored to compare our results [37]. Collectively, we observed excellent conservation of Dsl1 subunits in the opisthokont, archaeplastid, CCTH, and SAR clades, but very poor conservation in the amoebozoan and excavate lineages included. Strikingly, we failed to identify any subunits in *Entamoeba histolytica* and in *Trichomonas vaginalis*, and failed to identify any subunits outside of the SNARE Sec20 in *Leishmania major*, *Trypanosoma brucei*, and in *Giardia intestinalis*. Perhaps most importantly, we identified NAG/Sec39 homologues across Eukaryotes, despite Sec39 having been previously listed as fungal-specific [37]

### CORVET subunits Vps3 and Vps8 are ancient and related to HOPS

The HOPS and CORVET complexes share a single core of Vps11, Vps16, Vps18, and Vps33, each typified by two unique additional subunits [67,68]. The CORVET complex is defined by the additional subunits Vps3 and Vps8, and interacts with Rab5/Vps21 to direct tethering at endosomal compartments [69]. As Vps3 and Vps8 were not included in the previous comparative genomic study, and have only been studied extensively in yeast, we sought to determine whether they represent conserved eukaryotic machinery. We identified homologues of Vps8 in 11 of 12 opisthokont genomes, and in 26 of 31 genomes overall (Figure 1). Importantly, as with Tca17, we identified at least one organism with a Vps8 homologue from each supergroup, suggesting wide retention and an ancient origin.

**Figure 2:**
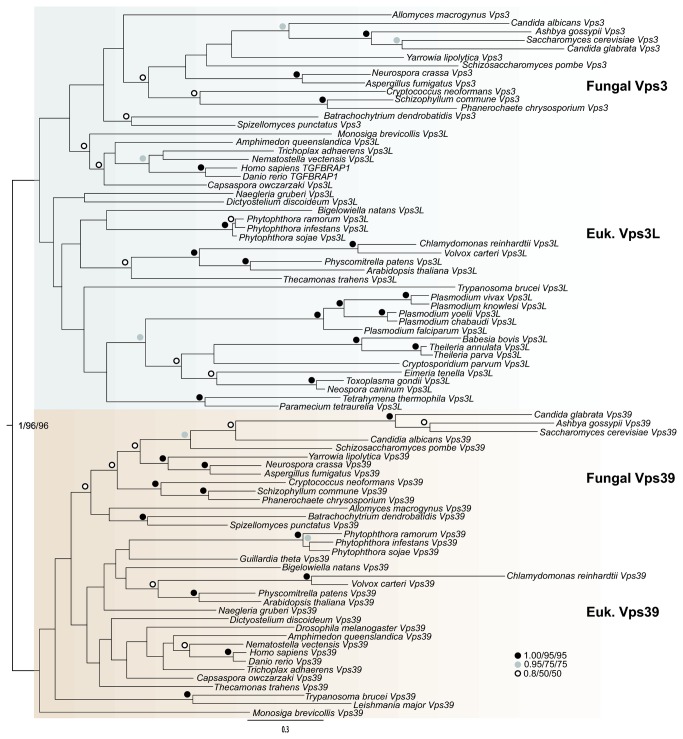
Phylogenetic analysis separating Vps3 family proteins from Vps39. This figure demonstrates that the duplication into Vps3 and 39 occurred early in eukaryotic evolution and that both are widely retained proteins. The best Bayesian topology is shown and values for critical nodes are shown in the order of Bayesian posterior probabilities, PhyML bootstrap and RAxML bootstrap values. All other nodes are symbolized as inset.

Initially, we could only identify putative Vps3 homologues in the fungal taxa under study, suggesting Vps3 may be a fungal-specific component of the CORVET complex. Vps3 and Vps39 have been proposed to be homologous, and share significant similarity, especially in their C-termini [69]. It is in this region that both are hypothesized to interact with Vps11, and hence mediate subunit exchange and the interchange of complex identity [70]. It seemed possible then, that Vps3 could be a fungal-specific duplication of the broadly conserved Vps39 subunit. To test this hypothesis, we broadened our taxonomic sampling to include several fungi as well as three other opisthokont taxa (see Methods). In order to identify putative Vps3 and Vps39 homologues in these taxa, we built and used HMMs of all initially identified homologues. We identified multiple Vps39-like sequences in several taxa, and at least one Vps3 homologue in each fungal lineage. Phylogenetic analysis was then performed on the entire dataset (Figure 2).

Our phylogenetic reconstruction separated two groups of sequences around a central node with excellent support values for all three methods employed (1/96/96). Fungal Vps3 and Vps39 sequences partitioned into the two groups as expected, but surprisingly, so did sequences from a number of other taxa. Notably, sequences from the Holozoa in the Vps3-like clade clustered around *H. sapiens* transforming growth factor beta-receptor associated protein-1 (TGFβRAP1), suggesting a potential role for this protein in CORVET function. The two human Vps39-like proteins are described by various nomenclature, including TRAP-1 and TLP, and other studies have suggested the possibility of both being involved in metazoan endolysosomal trafficking [71]. Our results are consistent with this notion, and further suggest that TGFβRAP1/TRAP-1 functions in CORVET, while hVps39-1/TLP functions in HOPS. A further insight gleaned from this tree is the divergent nature of Vps3 and Vps39 homologues across eukaryotes, evident from the lack of support for a number of internal nodes and the frequency of long branches. This suggests that there is a large degree of plasticity in these sequences, consistent with previous studies and our qualitative observations during the alignment process.

**Figure 3:**
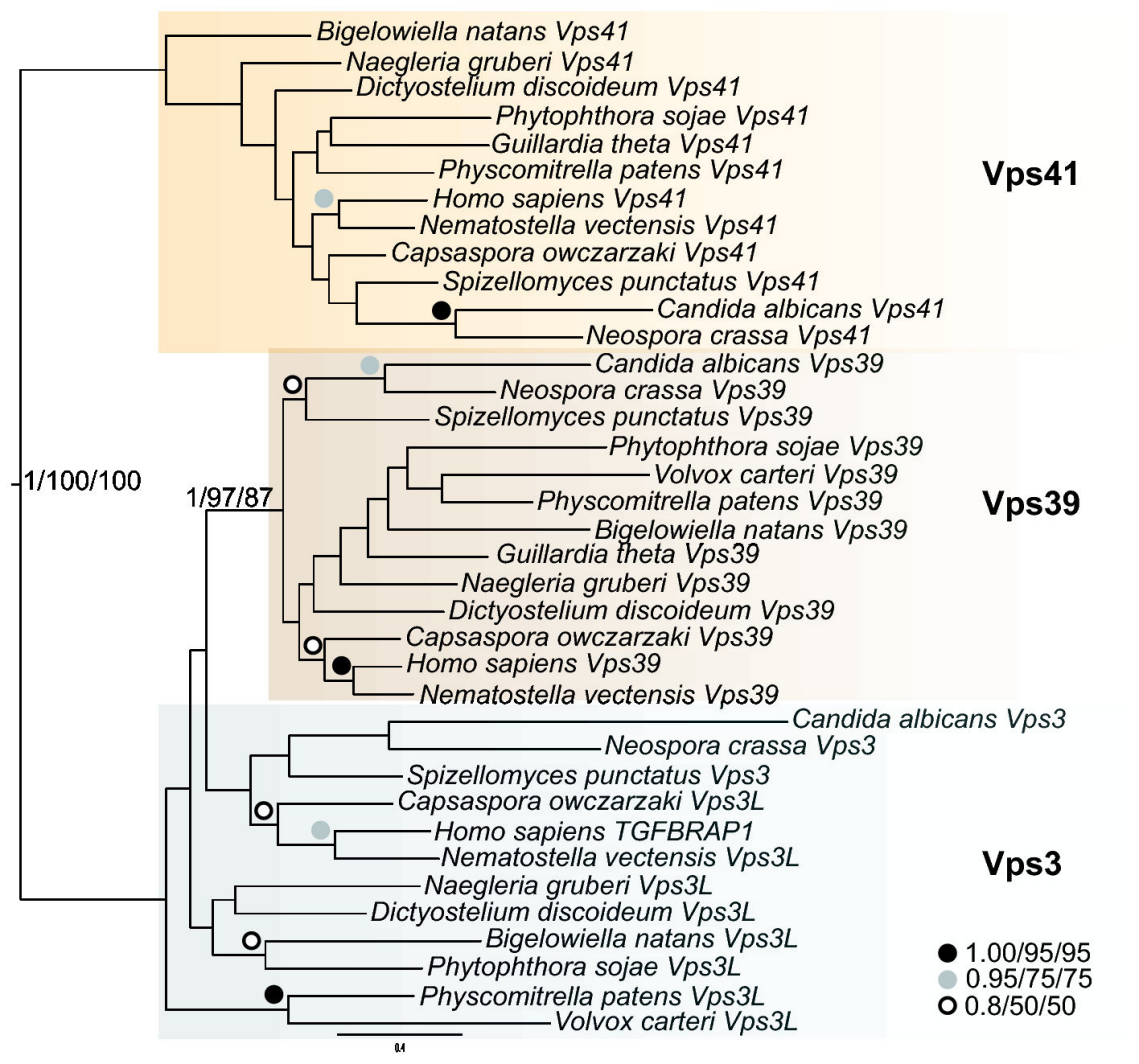
Phylogenetic analysis of Vps3, Vps39, and Vps41 proteins from select taxa. This figure demonstrates the separation of select Vps proteins from taxa spanning the breadth of eukaryotic diversity into distinct clades. Colour-coding is arbitrary and for visual purposes only.

Our HMM searches to identify the sequences used in Figure 2 revealed yet another potential aspect of the evolution of Vps3 and Vps39. We frequently retrieved homologues of Vps41 as the next best hit after Vps39-like sequences in a broad sampling of organisms. This is consistent with the homology of these proteins previously reported based on structural [70] and molecular evolutionary evidence [37]. In order to further validate the assigned orthology of our candidate Vps3 and Vps39 sequences, we chose a smaller sample of our large taxon set, and built trees including all Vps39-like sequences in addition to identified Vps41 homologues (Figure 3). We observed separation of the sequences into well-supported groupings of Vps41, Vps39-like, and a paraphyletic Vps3. This result confirms the identity of our putative Vps3 sequences, as they do not fall into the Vps39 clade, and are also distinct from the next most closely related group of sequences.

### Comparative genomic analysis of MTC components in Apicomplexa and their immediate outgroups

**Figure 4:**
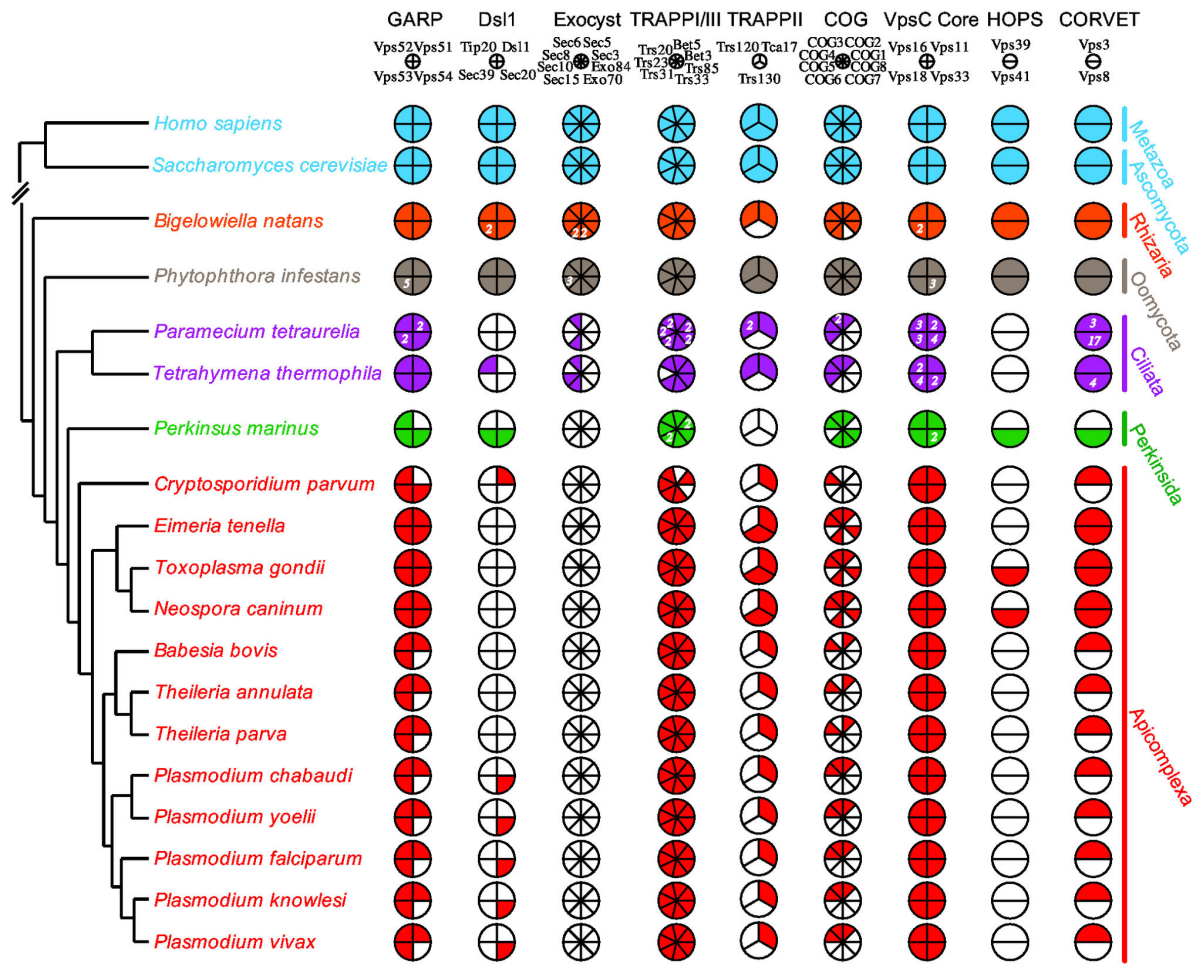
Comparative genomic survey of the eight MTCs across apicomplexans and select outgroups. This figure illustrates the sculpting of the MTC complement in alveolates and particularly in Apicomplexa across nearly all complexes. Coulson plot is read identically to Figure 1, and numbers in filled circles represent number of paralogues where appropriate. Colour-coding is not related to that found in Figure 1. The relationship between taxa are shown to the side, based on [114].

Having established what composed the ‘complete’ eukaryotic MTC complement, and that this was likely present in the ancestral lineage leading up to alveolates, we undertook a specific analysis of twelve apicomplexan genomes, as well as seven other genomes to function as outgroups in a nested hierarchy (Figure 4). These outgroup taxa included the well-studied organisms *H. sapiens* and *S. cerevisiae* as very distantly related organisms, where the vast majority of functional characterization of MTCs has taken place. Within the SAR clade, the rhizarian *Bigelowiella* *natans* and the stramenopile Phytophthora infestans were included as relatively distant taxa. Within the Alveolata, of which the Apicomplexa are members, we included two ciliates, Paramecium tetraurelia and Tetrahymena thermophila, and the more closely related dinoflagellate *Perkinsus marinus*. Results of the analysis are described by complex below.

#### The GARP complex is well conserved across Apicomplexa

GARP is an ancient tetramer composed of Vps51/Ang2, Vps52, Vps53, and Vps54 [37,72]. The complex functions to tether endosomal vesicles at the TGN, and likely plays a role in the assembly and stabilization of SNARE complexes [31,73]. Depletion of GARP subunits in mammalian cells results in endosome to TGN recycling defects, and subsequent mis-sorting of lysosomal hydrolases due to a lack of cation-independent mannose 6-phosphate receptors (CI-MPR) at the TGN [74]. GARP was conserved in all five non-apicomplexan lineages, though *P. marinus* appears to lack Vps51. Within the Apicomplexa, loss of Vps54 was most common, occurring in all five Plasmodium species, *B. bovis*, *T* *. annulata* , and *T* *. parva* . Homologues of all subunits with the exception of Vps51 were identified in *C. parvum*, while *T. gondii*, *E* *. tenella* , and *N* *. caninum* genomes encode putative homologues of all subunits.

#### DSL1 and exocyst complexes are mostly or entirely absent in Apicomplexa

In contrast to the relative conservation of the GARP complex, two MTCs were almost entirely absent in the alveolate genomes that we examined. All four subunits of the Dsl1/Syntaxin 18 complex were found in the opisthokont taxa, as well as *B* *. natans* and P. infestans. However, almost no subunits could be detected in any of the alveolate taxa included. With the exception of Sec20 homologues among Plasmodium species, and the lone Dsl1/ZW10 homologue in *C. parvum*, Apicomplexa completely lack this complex. *P. marinus*, with two subunits, still lacks Dsl1/ZW10, the main point of interaction between the complex and vesicles.

The loss of all eight exocyst subunits in all apicomplexan lineages, as well as in *P. marinus*, represents, perhaps, the most shocking result obtained in our searches. Composed of Sec3, Sec5, Sec6, Sec8, Sec10, Sec15, Exo70, Exo84, exocyst is involved in numerous and diverse cellular processes involving polarized exocytosis [75–78]. The total loss of exocyst in dinoflagellates and Apicomplexa, combined with the pattern of conservation in the ciliate lineages, suggests that loss of Sec3, Sec5, Exo70, and Exo84 may be synapomorphic for the alveolates, though a much broader taxon sampling would be required to support or refute this possibility.

#### MTCs reduced in Apicomplexa

All TRAPP complexes are built off the core TRAPPI complex, consisting of two copies of Bet3, as well as single copies each of Bet5, Trs20, Trs23, Trs31, and Trs33 [56,79]. Trs85, previously classified as part of the TRAPPI complex, was recently shown to be the defining member of a TRAPPIII complex [56,80]. TRAPPII contains the additional components, Trs120, Trs130, Trs65 and Tca17. All of these, with the exception of the fungal-specific Trs65, represent ancient complexes and thus are expected to be present in the ancestor of Apicomplexa. In yeast, TRAPPI mediates ER to Golgi transport, consistent with its activity as a Ypt1/Rab1 GEF [57,81]. Bet3, an important factor in GEF activity, is symmetrically distributed on either end of the complex and mediates interactions with the COPII coat through Sec23 [82]. Consistent with these important functions, Bet3 homologues were found in all lineages studied. Additionally, Trs20, Trs31, and Trs33 were identified in all taxa. The loss of various other subunits, including Bet5 and Trs85 in *C. parvum*, and Trs23 in *T* *. thermophila* may represent real subunit loss or incompleteness of the genomic databases.

Unlike the case with TRAPPI, TRAPPII functions are more controversial. Mutations in Trs120 and Trs130 result in defects in both endosome to Golgi traffic, as well as retrograde traffic through the Golgi [83]. Consistent with this, TRAPPII has been shown to interact with the gamma subunit of the COPI coat [84]. However, novel interactions between TRAPPC9/Trs120 and the p150 glued subunit of dynactin have also been reported [84], suggesting that TRAPPII may also play a role in the movement of COPII vesicles that have already been tethered at ERGIC and/or the cis-Golgi [85,86]. Some evolutionary lability of Trs130 is suggested by the results of previous studies [37] and the result that it was identified in three of the four non-alveolate outgroups. Consistent with this, we only found Trs130 in three of the 15 alveolates that we examined. However, Tca17 and Trs120 are conserved in the outgroup taxa, consistent with previous analyses ( [37] and Figure 1). Surprisingly, while we did identify Tca17 in all *Plasmodium* species, and most other Apicomplexa, we failed to identify Trs120 homologues in all apicomplexan lineages studied, as well as *P. marinus*. Indeed, we failed to identify homologues of any TRAPPII subunits in *P. marinus*. Differences between TRAPP organization and function between well-defined systems such as mammals and yeast further complicate any hypotheses regarding the conservation of TRAPP subunits in these lineages. Regardless of the actual organization of TRAPP complexes in the Apicomplexa and related taxa, however, it is clear that overall TRAPP function is reduced.

COG is an octameric complex, composed of two “lobes” (A and B) of four subunits each, connected to varying degrees in yeast and mammalian cells through the Cog1 and Cog8 subunits [87,88]. The main function of COG is to maintain proper glycosylation of proteins in the Golgi stack, through the continuous retrograde transport of relevant enzymes from endosomes to the TGN and within the Golgi itself [87,88]. In our comparative genomic analysis we found that subunit distribution was sparse. No alveolate lineage studied possessed Cog1, and almost all Apicomplexa additionally lacked Cog5-8. Conservation in *B. bovis*, *T* *. annulata* , *T* *. parva* , and *C. parvum* was especially lacking, with *C. parvum* encoding just Cog4 and the other three encoding Cog4 and Cog2. *T. gondii*, *N* *. caninum* , and *E* *. tenella* possess homologues of Cog6, and concurrent with this pattern of subunit retention, also possess a copy of Cog8, which may function to link Cog2-4 to Cog6 to form a fully functioning complex.

The CORVET/HOPS complex mediates tethering in the endolysosomal system. CORVET interacts with Rab5 at the early endosome, with HOPS taking over functions as the organelle matures into late endosomes and lysosomes, interacting with Rab7 and AP3 [71,89]. The complexes share a structural core of four subunits (Vps 11, 16, 18, 33), with the complex-specific additional subunits of HOPS (Vps39/ 41) or CORVET (Vps3/8) respectively providing interaction faces for the organellar markers. Consistent with the view of coordinated complex/organelle maturation, hybrid complexes have been reported to function at low levels in some cell types, composed of the core subunits and either Vps3/41 or Vps39/8 [69–71]. Based on the above analysis establishing the expected set of HOPS/CORVET subunits present in the ancestor of alveolates, we searched for the various subunits of the complexes. We identified homologues of all four Vps-C core subunits in all taxa under study. Multiple paralogs of all four subunits in P. tetraurelia, and of three of the four subunits in *T* *. thermophila* , may be indicative of life-stage or environmentally-regulated alternative complexes, or might simply be present due to the assembly of the macronuclear genomes of these organisms. Out of all organisms studied, the ciliate lineages most often exhibited paralagous gene expansion, including the notable presence of seventeen copies of putative Vps8 genes in P. tetraurelia. We observed a complete conservation of the structural core subunits in all apicomplexan taxa examined. Strikingly, Vps3 is also retained in all taxa, while Vps39, which occupies the same structural position in the complex, was not identified in any of the apicomplexans examined. Furthermore, the subunits occupying the structural position on the opposite face of the complex were either both missing, or in *Toxoplasma* and Neospora, both present. *E* *. tenella* represented the only exception, possessing a putative Vps8 gene, but no discernable copy of Vps41.

### MTC subunits are expressed

**Figure 5:**
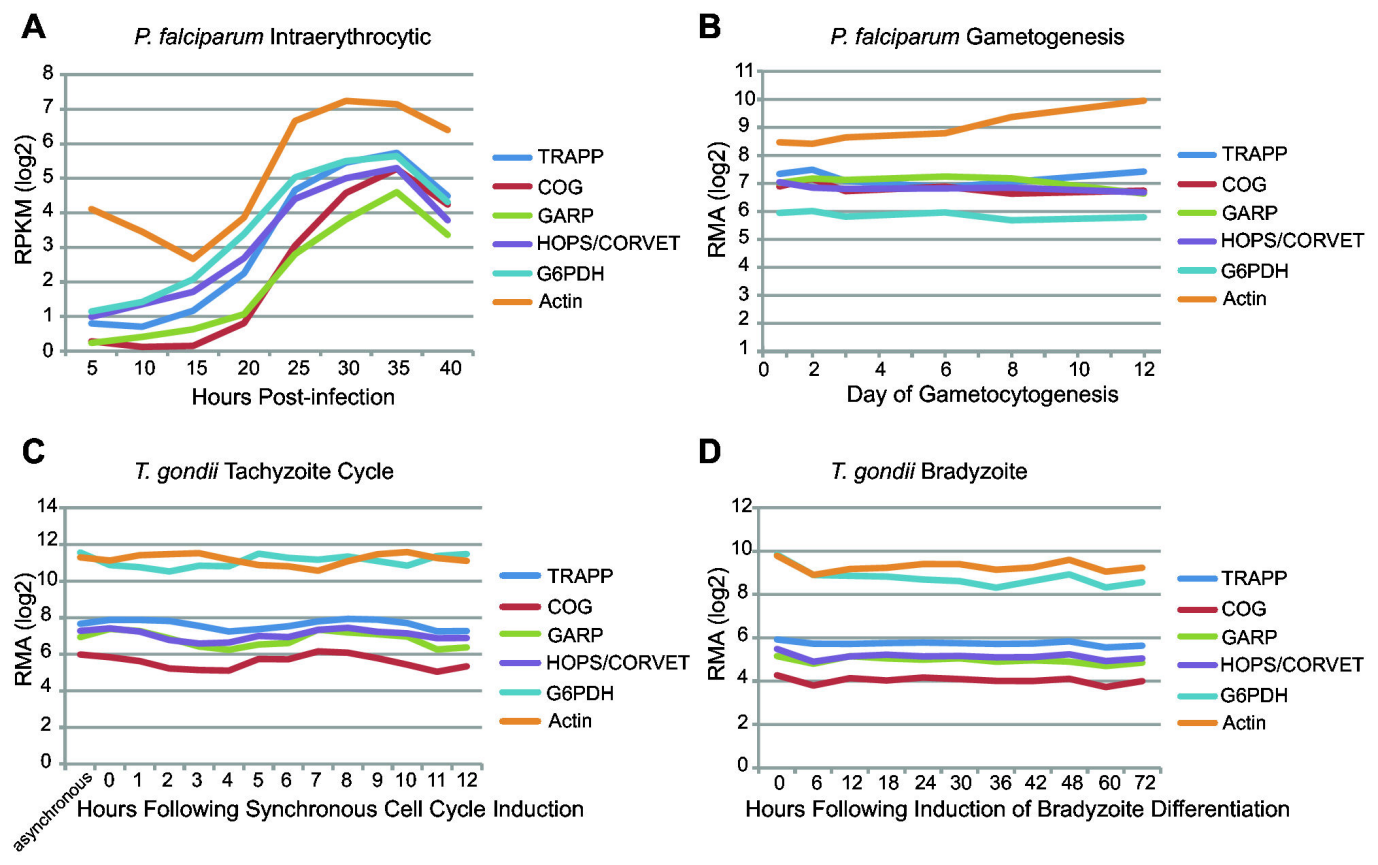
Average level of transcription of MTC subunits in *P. falciparum* and *T. gondii*. Data is provided for different lifecycle stages, and compared to the housekeeping genes Actin and G6PDH. As transcription data for each subunit of an MTC showed similar trends, the average of all subunits for each MTC is shown (see Figure S2). In all cases, tethering complex genes are not only transcribed, but have approximately equal levels of transcription and vary with time in a similar nature. This provides evidence for the continued regulation of transcription of these genes. All data was obtained publically from EuPathDB.

The reduced state of the some apicomplexan MTCs leads to two possible explanations. Either the complexes have been functionally disabled and are in the process of reductive evolution to complete loss, or their reduction represents a functional state, dependent on the unique biology of apicomplexan membrane trafficking. The latter hypothesis would predict the retained complex subunit genes being expressed in vivo. To this end, we utilized transcriptomic data for *P. falciparum* and *T. gondii*, available publically from EuPathDB, to determine if the subunits retained are expressed. Figure 5 shows the expression of each MTC as an average of the individual subunits compared to the housekeeping genes G6PDH and actin. This approach of averaging across complex subunits is justified; as all the subunits within a complex followed identical patterns of expression, and were similar in terms of the magnitude of expression (Figure S2). The general patterns that emerge are quite informative. The expression of each MTC follows a similar pattern that is unique to the organism and lifecycle stage. In addition, the magnitude of expression is generally lower than for both housekeeping genes. Hence, it is clear that MTC subunit genes are still expressed in these organisms. Consistent with this, proteomic data, also available publically from EuPath, confirms that the genes are not only being transcribed but translated to proteins (Table S2).

## Discussion

Here, we have undertaken a bioinformatic analysis of the MTC subunits in eukaryotic genomes. Building on previous analyses that established the base conservation of the HOPS, COG, exocyst, TRAPPI, TRAPPII, Dsl1 and GARP complexes as ancient, we have analyzed several newly reported subunits and complexes. We also specifically honed in on genomes of lineages leading up to and including the Apicomplexa and determined their MTC complement. These have provided us with data that can be used both to infer points of existing biology, as well as deduce the evolution of the MTCs.

The TRAPPII/III complexes had previously been proposed as ancient components of the membrane-trafficking system. Adding to this machinery, the Tca17 protein also appears to have been a part of the TRAPP machinery in the LECA. The Dsl1 complex had been reported likely to be ancient, but with a relatively sparse distribution. By contrast, our results suggest that Dsl1 is quite well conserved across eukaryotes. The human homologue of Dsl1, Zw10, was first identified as a kinetochore related protein [90] that, together with ROD and Zwilch, composes the RZZ complex involved in mitotic checkpoint control [91–93]. In concordance with our findings, an independent comparative genomic study identified diverse organisms containing both sets of Zw10 interacting proteins (those for the RZZ and NRZ/Syntaxin 18 complexes), and, interestingly, observed some correlation between the presence of the RZZ components, whether mitosis is open or closed, and the presence or absence of flagellae [62]. Finally, the CORVET complex had to this point never been examined in a broad eukaryotic survey. We can here report that this complex is also broadly conserved, bringing the total number of MTCs in the LECA to eight.

The Apicomplexa maintain a highly specialized, and in many ways divergent, set of membrane-trafficking machinery. The MTC complement, particularly in Apicomplexa, may in part be explained in light of this machinery. Dsl1 is the only MTC yet known to regulate the fusion of COPI vesicles at the ER, and may also have a role in preventing back fusion of ER-derived COPII vesicles (reviewed in 94). Given this, it is somewhat surprising that the Dsl1 complex was almost completely undetectable in the alveolate genomes we examined and may indicate an alternative mechanism for this presumably universal eukaryotic trafficking function. By contrast, the distribution of Dsl1 appears to correlate well with the presence of peroxisomes. Apicomplexa are the only major eukaryotic lineage to retain mitochondria but lack observable peroxisomes, and consistent with the microscopic data, they also show a near complete lack of the associated PEX machinery [15,95]. This lack of peroxisomes correlates well with the complete lack of the three specific Dsl1 components, with the SNARE Sec20 being present in some taxa. This last protein, however, acts as a general trafficking protein. Indeed the correlation of reduced or absent Dsl1 complexes and missing peroxisomes holds in our broad eukaryotic dataset as well (Figure 1). *Trichomonas* and *Entamoeba* lack all four Dsl1 proteins, while *Giardia* possesses the SNARE Sec20 and *Encephalitozoon* encodes only a single subunit. Even organisms with divergent peroxisomes show a reduced Dsl1 complement. The kinetoplastids do not possess regular peroxisomes but highly modified organelles entitled glycosomes, while the peroxisomes of diatoms have been shown to be at least somewhat reduced [15,96].

The reduced complement of COG and to some extent TRAPPII subunits could be reflective of the reduced Golgi morphology and unusual Golgi function in many Apicomplexa. We note that the organisms with the most complete sets of these complexes are indeed those with the most canonical Golgi bodies, at least morphologically. However, the TRAPPI/III subunits, also associated with Golgi, appear to be fully or nearly fully conserved. The functional implications of this will be interesting to pursue.

*T. gondii*, *P. falciparum*, and *C. parvum* all possess pathways for GPI anchor synthesis, as well as both O- and N-linked glycosylation [97–99]. Consistent with this, Cog2-4 were well conserved, as these subunits make up the bulk of lobe A, and perturbation of them in model systems produces severe growth and glycosylation defects, contrasting the relatively mild phenotypes of their lobe B counterparts [100]. Cog4, the only subunit universally conserved, is also the site of association of the COG complex with syntaxin5 and the Sly1 SM protein [101,102], both of which have homologues in Plasmodium, at least [37,103]. It is likely that all Apicomplexa possess less complex, though functional COG complexes. The results in *C. parvum* are somewhat troublesome, as an organism for which glycosylation activity has been studied, but that only appears to possess Cog4.

Particularly striking is the complete lack of exocyst subunits in all 12 apicomplexan genomes sampled. The roles of exocyst in model systems are diverse, but all involve the regulated movement of secretory vesicles. Given this, we propose that a bona fide absence of the complex could be explained by the strict positioning of the rhoptries and micronemes in the apical position of the cell, followed by a massive exocytic event that discharges their contents. This, combined with the apparent random positioning of the dense granules for secretion during the modification of the parasitophorous vacuole, would mean that the directed secretion that exocyst controls is no longer necessary and thus the complex could be lost. We would note, however, that the reduced selection for exocyst likely began before the strictly parasitic niche of Apicomplexa since not only could we not find any of the 8 subunits in Perkinsus, but we also noted a paucity of exocyst subunits in the ciliates as well. Though both ciliates lack the important subunits Sec3, Sec5, Exo70, and Exo84, they do encode the Sec6 subunit. Interaction between Sec6 and the plasma membrane SNARE Sec9 prevent SNARE complex assembly, but requires regions of Sec6 required for interaction with other exocyst subunits [104,105], and therefore some control over exocytosis may still be achieved by the remaining exocyst subunits in these lineages. The reduction of exocyst may well have been a pre-adaptation to the apicomplexan lifestyle rather than an evolutionary consequence thereof.

Several lines of evidence link the invasion organelles in Apicomplexa (i.e. rhoptries, micronemes and dense granules) with endocytic organelles such as endosomes and lysosomes [106,107]. Proteins trafficked to the rhoptries involve the action of the normally endocytic Rab5 [22], adaptin 1 and sortillin [24] and a microneme proteome identified several proteins generally associated with recycling endosomes including Rab11 and syntaxin 13. Previous work has identified specific reductions of endocytic machinery in Apicomplexa, such as the loss of AP3 [33] and of ESCRT complexes I and II [34], that have been speculated to be associated with modification of their endocytic organelles to their highly specialized role in invasion. Intriguingly, we observed a correlation with the retention pattern of the VpsC and accessory CORVET/HOPS subunits. Given the full conservation of the VpsC core and Vps3, as well as the evidence for expression, we are confident that a modified VpsC complex is functioning. It is tempting to speculate the existence of a single complex that functions in trafficking material to the micronemes and rhoptries, using a Rab5 mediated pathway, consistent with the full conservation of the Rab5 interactor Vps3 and the recent evidence for Rab5 involvement in this process [22]. The presence of both Vps41 and Vps8 in *Toxoplasma* and *Neospora* suggests that these taxa possess a more canonical, and possible dual functioning set of VpsC complexes. The Vps41 data correlates with the presence of AP3 in these taxa [33], albeit requiring the assumption that the highly divergent AP3 subunits in Plasmodium are indicative of loss in progress. Although challenging, the experimental testing of this hypothesis will be exciting and important to pursue.

In the case of several of the MTCs in Apicomplexa we observed reduction of the protein set but were able to identify some of the subunits. The transcriptomic data analyzed, supported by the proteomic data from *Toxoplasma* , shows that these genes are expressed and certainly suggests that the complexes are functional. If the complexes are functional in the taxa examined, then either they must be working as a minimal complex or using analogous subunits, i.e. proteins that serve the same function as the more canonical eukaryotic subunits but which are evolutionarily unrelated. Examples of analogous or species-specific subunits have been reported in other taxa, for instance, the recently described large complement of kinetoplastid-specific clathrin interacting proteins [108]. The latter possibility suggests that either MTC function can be maintained in reduced complexes, or that Apicomplexa possess novel interacting partners for the conserved subunits that modulate the specific trafficking necessary for their parasitic lifestyle. The possibility of apicomplexan specific subunits would raise exciting potential opportunities for therapeutics. Indeed, we have raised several hypotheses regarding MTC function in apicomplexans. These, however, would all need to be pursued functionally. In aid of this goal, our main analysis (Figure 4), produced curated annotations for a total of 388 predicted proteins. Of these, only 47 of 233 proteins in the Apicomplexa, and 68 of 155 proteins in the outgroup taxa had prior annotations consistent with our manually curated findings. Additionally, we newly identified homologues of ten proteins in the Apicomplexa, and two in outgroup taxa for which no gene models existed previously (see Table S2).

Despite the apparent sculpting of MTCs, and indeed multiple other trafficking factors, in the Apicomplexa, it is important to realize that many of these features may not represent modifications as a result of parasitism, but rather represent modifications in the ancestor of these lineages that were subsequently retained in parasitic lineages [109]. Early branching lineages of Apicomplexa and dinoflagellates, including the colpodellids, chromerids, perkinsids, and recently described Psammosa spp. share structures reminiscent of the apical complex in parasitic Apicomplexa [110–112]. Indeed, in this study, *P. marinus* often demonstrated patterns of retention closer to that of Apicomplexa than other outgroup taxa. Furthermore, recent studies suggest that a great diversity of early branching apicomplexan lineages exist globally, offering extensive opportunities to investigate the evolution of parasitism in the Apicomplexa [113]. To this end, the sequencing and annotation of genomes from the colpodellids, chromerids, and early branching dinoflagellates will be of great interest to the field moving forward toward an understanding of membrane-trafficking organelles in Apicomplexa, how they arose and what they are doing in some of the world’s most deadly parasites.

## Supporting Information

Figure S1 **Multisubunit tethering complexes encoded by the *Guillardia* *theta* and *Bigelowiella* *natans* nuclear genomes.** Most tethering complex components are well conserved in these two genomes. Filled sectors indicate the presence of a protein, empty sectors indicate that a protein was not identified, and numbers on filled sectors indicate multiple paralogues of a protein. Subunits are named according to *S. cerevisiae* nomenclature. Data are based on the results of BLAST and HMMer searches. Protein ID numbers are listed in Table S2.
(PDF)

Figure S2 **Transcriptome profiles for MTCs in *P. falciparum* and *T. gondii*.** Similar to Figure 5, data are presented for two different life stages in each organism. Large graphs represent A) *P. falciparum* intraerythrocytic cycle, B) *P. falciparum* gametogenesis, C) *T. gondii* tachyzoite cycle, and D) *T. gondii* bradyzoite differentiation expression of all MTCs for which expression data was available by subunit. The single large graph in each panel allows comparison of subunit expression between complexes, while the four small graphs facilitate intra-complex subunit comparisons for TRAPP, GARP, COG, and HOPS/CORVET respectively.
(PDF)

Table S1 **Genomes and databases analyzed.** Organisms are listed, along with strain information, database URL, and reference when applicable.
(DOCX)

Table S2 **Homologues identified in this study.** Data broken down by subunit and complex, with the exception of the complete analyses of G. theta and *B* *. natans* which were done as part of a genome analysis and are listed separately at the end of the table. For all cases, the organism searched, annotation, accession/data identifier, and E values for forward and reverse BLASTs, as well as HMMer searches are provided. The presence of supporting protomic data for *T. gondii* proteins is also listed and curation notes regarding domains and gene models are also listed when relevant.
(XLS)
