# Supplementary figures and images for: Comparative Genomic Analysis of Multi-Subunit Tethering Complexes Demonstrates an Ancient Pan-Eukaryotic Complement and Sculpting in Apicomplexa
Source: PLoS One. 2013 Sep 27;8(9):e76278. doi: 10.1371/journal.pone.0076278 (PMC3785458; doi:10.1371/journal.pone.0076278)

*Guillardia theta*

*Bigelowiella natans*

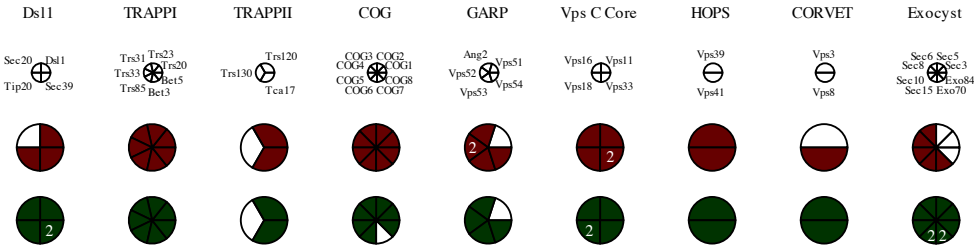

Supplement: Figure S1 — Multisubunit tethering complexes encoded by the Guillardia theta and Bigelowiella natans nuclear genomes. Most tethering complex components are well conserved in these two genomes. Filled sectors indicate the presence of a protein, empty sectors indicate that a protein was not identified, and numbers on filled sectors indicate multiple paralogues of a protein. Subunits are named according to S. cerevisiae nomenclature. Data are based on the results of BLAST and HMMer searches. Protein ID numbers are listed in Table S2. (PDF) [file pone.0076278.s001.pdf]
