# Supplementary material for: Comparative Genomic Analysis of Multi-Subunit Tethering Complexes Demonstrates an Ancient Pan-Eukaryotic Complement and Sculpting in Apicomplexa
Source: PLoS One. 2013 Sep 27;8(9):e76278. doi: 10.1371/journal.pone.0076278 (PMC3785458; doi:10.1371/journal.pone.0076278)
Supplement: Figure S2 — Transcriptome profiles for MTCs in P. falciparum and T. gondii. Similar to Figure 5, data are presented for two different life stages in each organism. Large graphs represent A) P. falciparum intraerythrocytic cycle, B) P. falciparum gametogenesis, C) T. gondii tachyzoite cycle, and D) T. gondii bradyzoite differentiation expression of all MTCs for which expression data was available by subunit. The single large graph in each panel allows comparison of subunit expression between complexes, while the four small graphs facilitate intra-complex subunit comparisons for TRAPP, GARP, COG, and HOPS/CORVET respectively. (PDF) [file pone.0076278.s002.pdf]

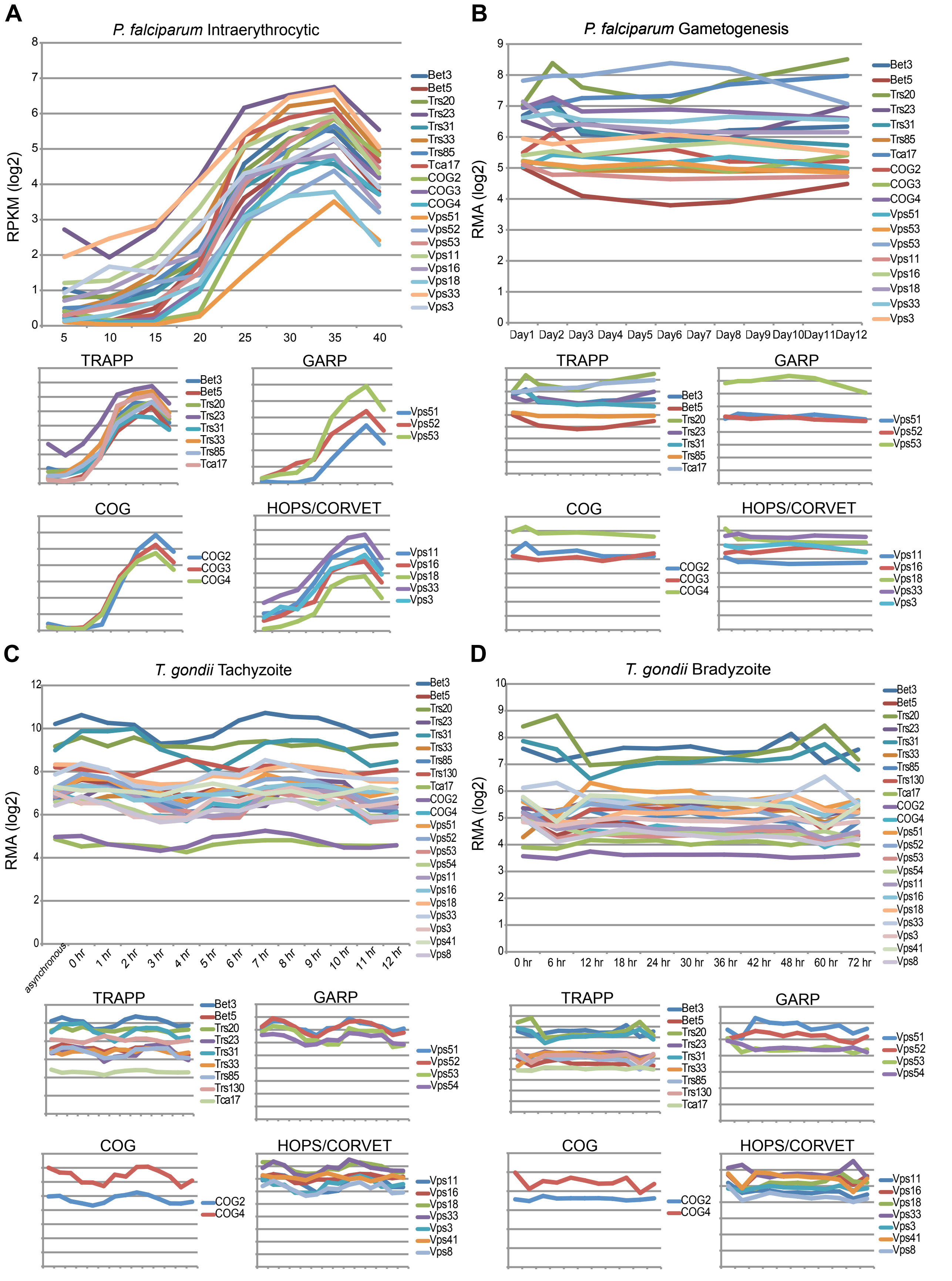
A
P. falciparum Intraerythrocytic
RPKM (log2)
5 10 15 20 25 30 35 40
Bet3
Bet5
Trs20
Trs23
Trs31
Trs33
Trs85
Tca17
COG2
COG3
COG4
Vps51
Vps52
Vps53
Vps11
Vps16
Vps18
Vps33
Vps3
TRAPP
GARP
COG
HOPS/CORVET
B
P. falciparum Gametogenesis
RMA (log2)
Day1 Day2 Day3 Day4 Day5 Day6 Day7 Day8 Day9 Day10 Day11 Day12
C
T. gondii Tachyzoite
RMA (log2)
asynchronous 0 hr 1 hr 2 hr 3 hr 4 hr 5 hr 6 hr 7 hr 8 hr 9 hr 10 hr 11 hr 12 hr
Trs130
Vps54
Vps41
Vps8
D
T. gondii Bradyzoite
RMA (log2)
0 hr 6 hr 12 hr 18 hr 24 hr 30 hr 36 hr 42 hr 48 hr 60 hr 72 hr
