# Supplementary material for: Comparative Genomic Analysis of Multi-Subunit Tethering Complexes Demonstrates an Ancient Pan-Eukaryotic Complement and Sculpting in Apicomplexa
Source: PLoS One. 2013 Sep 27;8(9):e76278. doi: 10.1371/journal.pone.0076278 (PMC3785458; doi:10.1371/journal.pone.0076278)
Supplement: Table S1 — Genomes and databases analyzed. Organisms are listed, along with strain information, database URL, and reference when applicable. (DOCX) [file pone.0076278.s003.docx]

| Organism and Strain | Database downloaded from | Reference |
| --- | --- | --- |
| *Allomyces macrogynus ATCC 38327* | <http://www.broadinstitute.org/> | Origins of Multicellularity Sequencing Project, Broad Institute of Harvard and MIT (http://www.broadinstitute.org/) |
| *Amphimedon queenslandica v1.0* | <http://www.ncbi.nlm.nih.gov/> | [The Amphimedon queenslandica genome and the evolution of animal complexity.](http://www.ncbi.nlm.nih.gov/pubmed/20686567) Srivastava M, et al. Nature 2010 Aug 5 |
| *Arabidopsis thaliana v8* | <http://www.phytozome.net/> | Swarbreck D, et al. [***The Arabidopsis Information Resource (TAIR): gene structure and function annotation.***](http://www.ncbi.nlm.nih.gov/pubmed/17986450) Nucleic Acids Res. 2008 Jan ; 36(Database issue):D1009-14. |
| *Ashbya gossypii ATCC 10895* | <http://www.ncbi.nlm.nih.gov/> | [The Ashbya gossypii genome as a tool for mapping the ancient Saccharomyces cerevisiae genome.](http://www.ncbi.nlm.nih.gov/pubmed/15001715) Dietrich FS, et al. Science 2004 Apr 9. |
| *Aspergillus fumigatus AF293* | <http://www.aspgd.org/> | [Nierman WC *et al.,*](http://www.ncbi.nlm.nih.gov/pubmed/16372009) "Genomic sequence of the pathogenic and allergenic filamentous fungus Aspergillus fumigatus.", *Nature*, 2005 Dec 22;438(7071):1151-6 |
| *Babesia bovis T2Bo* | <http://eupathdb.org/eupathdb/> | [**Genome Sequence of *Babesia bovis* and Comparative Analysis of Apicomplexan Hemoprotozoa**](http://www.plospathogens.org/article/info%3Adoi%2F10.1371%2Fjournal.ppat.0030148). Brayton KA, et al. (2007) Genome Sequence of *Babesia bovis* and Comparative Analysis of Apicomplexan Hemoprotozoa. PLoS Pathog 3(10): e148. doi:10.1371/journal.ppat.0030148 |
| *Batrachochytrium dendrobatidis JAM81* | <http://www.jgi.doe.gov/> | These sequence data were produced by the US Department of Energy Joint Genome Institute <http://www.jgi.doe.gov>/ in collaboration with the user community |
| *Bigelowiella natans CCMP2755* | <http://www.jgi.doe.gov/> | Curtis BA, et al. [Algal genomes reveal evolutionary mosaicism and the fate of nucleomorphs.](http://www.ncbi.nlm.nih.gov/pubmed/23201678) Nature. 2012 Nov 28. |
| *Candida albicans SC5314* | <http://www.candidagenome.org/> | Jones, T., et al. (2004) The Diploid Genome of *Candida albicans*. PNAS 101:7329-7334. |
| *Candida glabrata CBS138* | <http://www.candidagenome.org/> | Dujon et al., 2004, Nature 430:35-44; Koszul et al., 2003, FEBS Lett. 534(1-3):39-48 |
| *Capsaspora owczarzaki ATCC 30864* | <http://www.broadinstitute.org/> | Origins of Multicellularity Sequencing Project, Broad Institute of Harvard and MIT (http://www.broadinstitute.org/) |
| *Chlamydomonas reinhardtii v4* | <http://www.phytozome.net/> | Merchant SS, et al., [The Chlamydomonas genome reveals the evolution of key animal and plant functions.](http://www.ncbi.nlm.nih.gov/sites/entrez?Db=pubmed&Cmd=ShowDetailView&TermToSearch=17932292) Science. 2007 Oct 12;318(5848):245-50 |
| *Cryptococcus neoformans H99* | <http://www.jgi.doe.gov/> | These sequence data were produced by the US Department of Energy Joint Genome Institute <http://www.jgi.doe.gov>/ in collaboration with the user community |
| *Cryptosprodidium parvum IowaII* | <http://eupathdb.org/eupathdb/> | Abrahamsen, M. S., et al. (2004). **Complete genome sequence of the apicomplexan, Cryptosporidium parvum.** [Science.](http://www.ncbi.nlm.nih.gov/pubmed/15044751#) 2004 Apr 16;304(5669):441-5. |
| *Danio rerio Zv9* | <http://www.ncbi.nlm.nih.gov/> | The Danio rerio Sequencing Project (<http://www.sanger.ac.uk/Projects/D_rerio/>); Wellcome Trust Sanger Institute |
| *Dictyostelium discoideum* | <http://dictybase.org/> | Eichinger et al., The genome of the social amoeba *Dictyostelium discoideum,* 2005, Nature 435:43-57 |
| *Drosophila melanogaster* | <http://flybase.org/> | **The Genome Sequence of Drosophila melanogaster.** Mark D. Adams, et al. Science 24 March 2000: **287** (5461), 2185-2195. |
| *Eimeria tenella Houghton* | <http://eupathdb.org/eupathdb/> | Produced by the Parasite Genomics Group at the Wellcome Trust Sanger Institute. |
| *Emiliania huxleyi CCMP1516* | <http://www.jgi.doe.gov/> | These sequence data were produced by the US Department of Energy Joint Genome Institute http://www.jgi.doe.gov/ in collaboration with the user community |
| *Encephalitozoon cuniculi GBM1* | <http://eupathdb.org/eupathdb/> | Katinka MD, et al. **Genome sequence and gene compaction of the eukaryote parasite Encephalitozoon cuniculi.** [Nature.](http://www.ncbi.nlm.nih.gov/pubmed/11719806#) 2001 Nov 22;414(6862):450-3. |
| *Entamoeba histolytica HM1* | <http://eupathdb.org/eupathdb/> | Loftus B, et al. **The genome of the protist parasite Entamoeba histolytica.** [Nature.](http://www.ncbi.nlm.nih.gov/pubmed/15729342#) 2005 Feb 24;433(7028):865-8. |
| *Giardia intestinalis WB* | <http://eupathdb.org/eupathdb/> | Morrison HG, et al: **Genomic minimalism in the early diverging intestinal parasite Giardia lamblia.** *Science* 2007, **317:**1921-192610. |
| *Guillardia theta CCMP2712* | <http://www.jgi.doe.gov/> | Curtis BA, et al. [Algal genomes reveal evolutionary mosaicism and the fate of nucleomorphs.](http://www.ncbi.nlm.nih.gov/pubmed/23201678) Nature. 2012 Nov 28. |
| *Homo sapiens* | <http://www.ncbi.nlm.nih.gov/> | [International Human Genome Sequencing Consortium](http://www.ncbi.nlm.nih.gov/pubmed/15496913) "Finishing the euchromatic sequence of the human genome.", *Nature*, 2004 Oct 21;431(7011):931-45 |
| *Leishmania major Friedlin* | <http://eupathdb.org/eupathdb/> | Ivens AC, et al. **The Genome of the Kinetoplastid Parasite, *Leishmania major.*** [Science. 2005 July 15; 309(5733): 436–442.](http://www.ncbi.nlm.nih.gov/entrez/eutils/elink.fcgi?dbfrom=pubmed&retmode=ref&cmd=prlinks&id=16020728) |
| *Micromonas spp. RCC299* | <http://www.jgi.doe.gov/> | Alexandra Z. Worden, et al. [*Green Evolution and Dynamic Adaptations Revealed by Genomes of the Marine Picoeukaryotes Micromonas*](http://www.sciencemag.org/cgi/content/abstract/324/5924/268)[.](http://www.sciencemag.org/cgi/content/abstract/324/5924/268)) Science. 2009 April 10;324(5924):268-272 |
| *Monosiga brevicollis* | <http://www.jgi.doe.gov/> | King N, et al. [*The genome of the choanoflagellate*](http://www.ncbi.nlm.nih.gov/pubmed/18273011). Nature. 2008 Feb 14;451(7180):783-8. |
| *Naegleria gruberi v1* | <http://www.jgi.doe.gov/> | Jillian K. Fritz-Laylin, et al. [The Genome of Naegleria gruberi Illuminates Early Eukaryotic Versatility](http://www.cell.com/retrieve/pii/S009286741000067X) Cell, March, 2010 |
| *Nematostella vectensis* | <http://www.jgi.doe.gov/> | Putnam NH, et al. (2007) [*Sea anemone genome reveals ancestral eumetazoan gener epertoire and genomic organization*](http://dx.doi.org/10.1126/science.1139158). Science. 317, 86-94. |
| *Neospora caninum Liverpool* | <http://eupathdb.org/eupathdb/> | The genome of *Neospora caninum* Liverpool was produced by the Parasite Genomics Group at the Wellcome Trust Sanger Institute. |
| *Neurospora crassa* | <http://www.broadinstitute.org/> | Provided pre-publication by the BROAD Institute, in collaboration with the user community |
| *Ostreococcus tauri v2* | <http://www.jgi.doe.gov/> | Palenik B, et al. (2007) [*The tiny eukaryote Ostreococcus provides genomic insights into the paradox of plankton speciation.*](http://dx.doi.org/10.1073/pnas.0611046104) Proc Natl Acad Sci U S A., 104, 7705-7710. |
| *Paramecium tetraurelia d4-2* | <http://www.ncbi.nlm.nih.gov/> | Aury JM, et al. **Global trends of whole-genome duplications revealed by the ciliate Paramecium tetraurelia.** [Nature.](http://www.ncbi.nlm.nih.gov/pubmed/17086204#) 2006 Nov 9;444(7116):171-8. |
| *Perkinsus marinus ATCC 50983* | <http://www.ncbi.nlm.nih.gov/> | Provided pre-publication by the JCVI, in collaboration with the user community |
| *Phanerochaete chrysosporium v2* | <http://www.jgi.doe.gov/> | [Genome sequence of the lignocellulose degrading fungus Phanerochaete chrysosporium strain RP78.](http://dx.doi.org/10.1038/nbt967) Martinez D, et al. *Nature Biotechnology* **22**, 695 - 700 (2004) [DOI: 10.1038/nbt967] |
| *Physcomitrella patens v1.6* | <http://www.phytozome.net/> | Rensing SA, et al. [*The Physcomitrella genome reveals evolutionary insights into the conquest of land by plants*](http://www.ncbi.nlm.nih.gov/pubmed/18079367)*.* Science. 2008 Jan 4;319(5859):64-9. |
| *Phytophthora infestans T30-4* | <http://www.ncbi.nlm.nih.gov/> | Haas BJ, et al. Genome sequence and analysis of the Irish potato famine pathogen *Phytophthora infestans. Nature* **461**, 393-398. |
| *Phytophthora ramorum v1.1* | <http://www.jgi.doe.gov/> | Tyler BM, et al. (2006) [*Phytophthora Genome Sequences Uncover Evolutionary Origins and Mechanisms of Pathogenesis*](http://dx.doi.org/10.1126/science.1128796). Science. 313, 1261-1266. |
| *Phytophthora sojae v3* | <http://www.jgi.doe.gov/> | These sequence data were produced by the US Department of Energy Joint Genome Institute http://www.jgi.doe.gov/ in collaboration with the user community |
| *Plasmodium chabaudi chabaudi* | <http://eupathdb.org/eupathdb/> | The genome of *Plasmodium chabaudi* AS was produced by the Parasite Genomics Group at the Wellcome Trust Sanger Insitute. |
| *Plasmodium falciparum 3D7* | <http://eupathdb.org/eupathdb/> | Gardner et al., Nature 2002; 419:498-511 |
| *Plasmodium knowlesi H* | <http://eupathdb.org/eupathdb/> | Pain et al. Nature 2008; 455:799-803. |
| *Plasmodium vivax SaI1* | <http://eupathdb.org/eupathdb/> | Comparative genomics of the neglected human malaria parasite Plasmodium vivax.. Nature 2008 Oct 9;455(7214):757-63 Carlton et al. |
| *Plasmodium yoelii 17XNL* | <http://eupathdb.org/eupathdb/> | Carlton JM, et al. Genome sequence and comparative analysis of the model rodent malaria  parasite Plasmodium yoelii yoelii. Nature. 2002 October 3; 419: 512-519. |
| *Rhizopus oryzae 99-880* | <http://www.jgi.doe.gov/> | **Ma LJ, et al.** Genomic analysis of the basal lineage fungus Rhizopus oryzae reveals a whole-genome duplication. **PLoS Genet.** 2009 Jul;5(7):e1000549. Epub 2009 Jul 3. |
| *Saccharomyces cerevisiae S288c* | <http://www.ncbi.nlm.nih.gov/> | [Life with 6000 genes.](http://www.ncbi.nlm.nih.gov/pubmed/8849441) Goffeau A, et al. Science 1996 Oct 25 |
| *Schizophyllum commune v2* | <http://www.jgi.doe.gov/> | Ohm RA, et al. [*Genome sequence of the model mushroom Schizophyllum commune.*](http://www.ncbi.nlm.nih.gov/pubmed/20622885) Nature Biotech. 2010 Sep;28(9):957-63 |
| *Schizosaccharomyces pombe ASM294* | <http://www.pombase.org/> | Wood V, et al. The genome sequence of Schizosaccharomyces pombe. [Nature.](http://www.ncbi.nlm.nih.gov/pubmed/11859360#) 2002 Feb 21;415(6874):871-80. |
| *Spizellomyces punctatus DAOM BR117* | <http://www.broadinstitute.org/> | Origins of Multicellularity Sequencing Project, Broad Institute of Harvard and MIT (http://www.broadinstitute.org/) |
| *Tetrahymena thermophila* | <http://www.ncbi.nlm.nih.gov/> | [**Macronuclear Genome Sequence of the Ciliate *Tetrahymena thermophila,* a Model Eukaryote**](http://www.plosbiology.org/article/info%3Adoi%2F10.1371%2Fjournal.pbio.0040286). Eisen JA, et al. (2006) Macronuclear Genome Sequence of the Ciliate *Tetrahymena thermophila,* a Model Eukaryote. PLoS Biol 4(9): e286. doi:10.1371/journal.pbio.0040286 |
| *Thalassiosira pseudonana* | <http://www.jgi.doe.gov/> | [The Genome of the Diatom Thalassiosira Pseudonana: Ecology, Evolution, and Metabolism](http://www.sciencemag.org/cgi/content/full/306/5693/79). Armbrust EV, et al. Science. 2004 Oct 1;306(5693):79-86. |
| *Thecamonas trahens ATCC 50062* | <http://www.broadinstitute.org/> | Origins of Multicellularity Sequencing Project, Broad Institute of Harvard and MIT (http://www.broadinstitute.org/) |
| *Theileria annulata Ankara* | <http://eupathdb.org/eupathdb/> | Genome of the host-cell transforming parasite Theileria annulata compared with T. parva.. Science 2005 Jul 1;309(5731):131-3 Pain et al. |
| *Theileria parva Muguga* | <http://eupathdb.org/eupathdb/> | Genome sequence of Theileria parva, a bovine pathogen that transforms lymphocytes.. Science 2005 Jul 1;309(5731):134-7 Gardner et al. |
| *Toxoplasma gondii* | <http://eupathdb.org/eupathdb/> | Kissinger JC, et al. **ToxoDB: accessing the Toxoplasma gondii genome.** [Nucleic Acids Res.](http://www.ncbi.nlm.nih.gov/pubmed/12519989#) 2003 Jan 1;31(1):234-6. |
| *Trichomonas vaginalis* | <http://eupathdb.org/eupathdb/> | Draft genome sequence of the sexually transmitted pathogen Trichomonas vaginalis. Science 2007 Jan 12;315(5809):207-12 Carlton et al. |
| *Trichoplax adhaerens Grell-BS-1999* | <http://www.jgi.doe.gov/> | Srivastava M, et al. [The Trichoplax genome and the nature of placozoans.](http://www.nature.com/nature/journal/v454/n7207/abs/nature07191.html) Nature. 2008 Aug 21;454(7207):955-60. |
| *Trypanosoma brucei 927* | <http://eupathdb.org/eupathdb/> | The genome of the African trypanosome Trypanosoma brucei. Science 2005 Jul 15;309(5733):416-22 Berriman et al. |
| *Volvox carteri* | <http://www.phytozome.net/> | Prochnik, S. E., et al. (2010). Genomic analysis of organismal complexity in the multicellular green alga *Volvox carteri*. Science, 329(5988), 223-226. doi:10.1126/science.1188800 |
| *Yarrowia lypolytica CLIB122* | <http://www.ncbi.nlm.nih.gov/> | [Genome evolution in yeasts.](http://www.ncbi.nlm.nih.gov/pubmed/15229592) Dujon B, et al. Nature 2004 Jul 1 |
